# qSOFA score for prediction of sepsis outcome in emergency department

**DOI:** 10.12669/pjms.36.4.2031

**Published:** 2020

**Authors:** Kavous Shahsavarinia, Payman Moharramzadeh, Reza Jamal Arvanagi, Ata Mahmoodpoor

**Affiliations:** 1 Kavous Shahsavarinia, Associate Professor, Road Traffic Injury Research Center, Department of Emergency Medicine, Faculty of Medicine, Tabriz University of Medical Sciences, Tabriz, Iran; 2 Payman Moharramzadeh, Associate Professor, Department of Emergency Medicine, Faculty of Medicine, Tabriz University of Medical Sciences, Tabriz, Iran; 3 Reza Jamal Arvanagi, Emergency Medicine Specialist, Department of Emergency Medicine, Faculty of Medicine, Tabriz University of Medical Sciences, Tabriz, Iran; 4 Prof. Dr. Ata Mahmoodpoor, Department of Anesthesiology and intensive care, Faculty of Medicine, Tabriz University of Medical Sciences, Tabriz, Iran

**Keywords:** Emergency department, qSOFA ((quick sequential organ failure assessment), Sepsis

## Abstract

**Objective:**

The third international consensus definition for sepsis and septic shock (sepsis 3) task force recently introduced qSOFA (quick sequential organ failure assessment) as a score for detection of patients at risk of sepsis outside of intensive care units. We performed this study to evaluate the validity of qSOFA for early detection and risk stratification of septic patients in emergency department.

**Methods:**

We conducted this study in an emergency department of the largest university affiliated hospital in northwest of Iran from Sept 2015 to Sept 2016. One hundred and forty patients who were SIRS positive with a suspected infection without alternative diagnosis and a microbiological proven infection were enrolled in this study. qSOFA was calculated for each patient and correlated with sepsis grades and mortality.

**Results:**

From 140 patients 84 (60%) had positive qSOFA score and 56 (40%) patients had negative qSOFA score. Our results showed that near half of patients with positive qSOFA expired during their stay in hospital while this was about 5% for patients with negative qSOFA. ROC curve of study regarding prediction of outcome with qSOFA showed an area under curve of 0.59. (*P* value: 0.04). Time spent to sepsis detection was 16 minutes shorter with qSOFA score compared to SIRS criteria in this study.

**Conclusion:**

In patients with suspected sepsis, qSOFA has acceptable value for risk stratification of severity, multi organ failure and mortality. It seems that education of medical staff and frequent screening of patients for warning signs can help to increase the value of qSOFA in prediction of mortality in critically ill septic patients.

## INTRODUCTION

Sepsis is a huge global health problem and represents great challenge regarding diagnosis and treatment for physicians.^1^ Sepsis is a frequently encountered life threatening condition and is estimated that approximately eight hundred fifty thousand (850000) cases of sepsis visit emergency department annually which is associated with high cost.^2^ Sepsis is defined as life threatening organ dysfunction caused by dysregulated host response to infection.^3^ Organ dysfunction is characterized by acute increase in two or more points of sequential organ failure assessment (SOFA) score; but as it requires different tests and rarely measured outside the ICU, a new score, qSOFA (quick sequential organ failure assessment), is introduced by sepsis task force^3^.

Results of a recently performed cohort study showed that qSOFA was better than SIRS in predicting in-hospital mortality in septic patients in emergency department.^4^ On the other hand, data from New Zealand intensive care society showed that interventions like intubation, sedation and mechanical ventilation can interfere with the validity and accuracy of qSOFA score in the critically ill patients.^5^,^6^ Thus, SOFA remains the only score for prediction of sepsis in this setting. Rodriguez et al.(2018) showed that qSOFA is equal or better than SIRS (systemic inflammatory response syndrome) in predicting the critical illness in patients with sepsis admitted from ED.^7^ Results of two recently performed studies showed that although qSOFA may be valuable in predicting sepsis related mortality but it is a poorly sensitive marker for mortality prediction as it can delay the initiation of intervention known to improve sepsis related outcomes.^8^,^9^

Based on the above, there are some criticism about low sensitivity of qSOFA for prediction of outcome and resultant delayed diagnosis of sepsis and lack of endorsement by scientific societies and resultant mis-implication as a clinical decision tool.^10^-^12^ Churpek et al.(2017) showed that commonly used early warning scores are more accurate than qSOFA in prediction of mortality in septic patients outside the ICU.^13^ Results of two other studies showed that qSOFA had good prognostic value for mortality in septic patients in resource limited countries and supports using it as a triage tool to identify the patients at risk of poor outcome in resource limited countries.^14^,^15^ Based on the literature, it seems that qSOFA has been found as a contradictory marker for its diagnostic and prognostic value of sepsis evaluated so far. We performed this study to evaluate the diagnostic and prognostic value of qSOFA in septic patients admitted to emergency department.

## METHODS

After obtaining ethics committee approval (Ref. No.: 5/d/20365, dated April 19, 2018), one hundred and forty patients admitted to emergency department with sepsis diagnosis were included in this study. We performed a retrospective study on all patients transferred to emergency medicine department of Imam Reza hospital between 1^st^ Sept 2015 and 1^st^ Sep 2016. All patients with a suspected infection who were SIRS positive without alternative diagnosis and a microbiological proven infection were enrolled in this study. Exclusion criteria were patients with age less than 18 years old pregnant women, prisoners, severe traumatic patients, and patients with malignancy. Suspicion of infection was made using the following data extracted from hospital records: blood pressure, heart rate, body temperature, respiratory rate and level of consciousness. Demographic and laboratory variables were recorded for all patients. We calculated qSOFA for each patient. The score ranges from 0 to three with one point allocated for each of the following clinical signs: systolic blood pressure <100 mmHg respiratory rate > 22/minutes and altered mental status from baseline. A score of equal or more than two indicates more severity with increased ICU length of stay and mortality. The SIRS criteria use the clinical criteria of surviving sepsis campaign for severe sepsis^15^ with the presence of at least two of the followings: HR>90/min, RR>25/min. BT>38 or BT<36, WBC >12000 or less than 4000. We considered patients as sepsis if they had two or more than SIRS criteria with positive microbial cultures, severe sepsis if had sepsis with organ failure, septic shock if they need vasopressor to keep their systolic blood pressure more than 90 and multi organ dysfunction syndrome as septic shock with two or more organ failure. We performed an education course to all emergency department medical staff regarding qSOFA warning alarms one month before study enrollment.

### Statistical analysis

All data were expressed as mean ± standard deviation (SD) and analyzed using the Statistical Package for Social Sciences (version 20). We used students’ test for comparing variables. We used ROC curve analysis for prediction of outcome in patients. Specificity and sensitivity of qSOFA for outcome prediction were also calculated. P value of <0.05 was considered to be statistically significant.

## RESULTS

**Fig.1:**
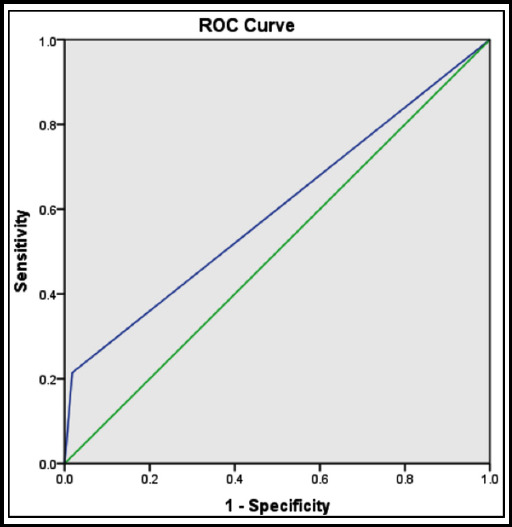
Prediction of outcome by qSOFA.

One hundred and forty patients were enrolled in this study. The mean value for their age was 59±16.1 and 45.7% of them were women. The mean age of both sex did not have a significant difference (*P*-value: 0.46). We divided our patients due to their severity of illness into four groups: sepsis, severe sepsis, septic shock and multi organ failure. Demographic characteristics of patients are shown in Table-I. From 140 patients, 84(60%) had positive qSOFA score and 56 patients had negative qSOFA score. During the study period, 121 patients were survived and 19 patients were expired. All of patients with septic shock and multi organ failure expired during their hospitalization. Among patients with sepsis and severe sepsis 98.7% and 89% survived respectively. Our results showed that 32% of patients with positive qSOFA were expired during their stay while this was about 5% for patients with negative qSOFA (Table-II). ROC curve of study regarding prediction of outcome with qSOFA showed an area under curve of 0.59. (*P*-value: 0.04) (Fig.1). In this study sensitivity of qSOFA for detection of sepsis was 66.3% with a specificity of 60.6%. Negative predictive value and positive predictive value for qSOFA in sepsis detection was 35.7% and 84.5%, respectively based on the clinical diagnostic statement by surviving sepsis campaign. Time spent to sepsis detection is 16 minutes shorter with qSOFA score compared to SIRS criteria in this study as this score didn’t need laboratory assessment. This is performed with the time duration between suspicion of sepsis and the confirmation of diagnosis.

**Table-I:** Demographic characteristics of patients.

Variable	Value
Sex M/F	76/64
Age	59±16.1
APACHE score	25.3±10.43
***Comorbidities***	
Diabetes	31
Hyperlipidemia	19
Hypertension	45
IHD/CHF	38
Kidney disease	8
Lung disease	17
Liver disease	2
Neurologic disorder	9

M/F: Male/Female, APACHE: Acute Physiologic and Chronic Health Evaluation.

IHD/CHF: Ischemic Heart Disease/Congestive Heart Failure

**Table-II:** qSOFA and patient’s outcomes

Variable	Value
***qSOFA +/-***	
Sepsis	39/40
Severe sepsis	32/16
Septic shock	10/0
MOF	3/0
***Mean Hospital stay (day)***	
Sepsis	5.74±2.58
Severe sepsis	9.36±3.89
Septic shock	5.6±1.83
MOF	2.66±1.52
***Outcome (survived)***	
Sepsis	78(98.7%)
Severe sepsis	43(89%)
Septic shock	0(0%)
MOF	0(0%)

## DISCUSSION

Results of this study showed that qSOFA score was associated with increased mortality and organ failure in septic patients. Thus, qSOFA is a good prognostic marker for mortality and multi organ failure in septic patients but is not a good diagnostic marker for sepsis detection. Our findings were similar to previous study in Malawy which showed that qSOFA is a simple tool that can aid risk stratification in resource-limited settings.^16^

Although there is huge evidence regarding optimal management and early detection of sepsis in ICUs, medical staff especially in emergency departments has an essential role in optimal management and early detection of these patients.^1^ So, all medical staff needs to know a simple and easy score to diagnose sepsis and detect the patients with poor outcome for appropriate management.^17^ Rudd et al. evaluated the validity of qSOFA in low middle income countries showed that among hospitalized patients with suspected infection, the qSOFA score compared to SIRS criteria better identified patients with high risk of death but predictive value differs among different centers/countries.^18^ Opposite to this results, Askim et al.^19^ showed that qSOFA failed to be a risk stratification tool to predict mortality in patients with severe sepsis because of low sensitivity. These results are in contrast with our results which showed only a good predictive role of qSOFA in prediction of mortality in septic shock patients but its predictive value in patients with sepsis and severe sepsis was low. Churpek et al. in a study recommended that qSOFA should not be used as a predictor marker for mortality in patients with suspected infection.^13^ Results of a recently performed meta-analysis defined that qSOFA is a poor sensitive predictor marker of in-hospital mortality in patients with suspected infection. However, the sensitivity of qSOFA was higher for patients in ICUs compared to the patients in other wards.^20^ So, based on the results of these studies and the importance of early detection and appropriate management of septic patients for their outcome, we should emphasis on the following subjects: 1) the pre-hospital and emergency triage based on clinical signs that are easy to recognize and implementation of recommended bundles, 2) the education and implementation of qSOFA score in all medical staff not only intensivists for early detection and appropriate treatment of critically ill patients. Results of another recently published meta-analysis^21^ defined that SIRS criteria were significantly superior to the qSOFA in diagnosis of sepsis, but the qSOFA was slightly better than the SIRS in predicting hospital mortality which is similar to our results. They recommended the combination of both criteria to produce a better model for initiation or escalation therapy in septic patients.^22^ Finkelsztein et al. showed that qSOFA had greater accuracy than SIRS criteria in prediction of ICU free days and mortality. However, results regarding ventilator free days and organ dysfunction free days were inclonclusive.^22^ Haydar and coworkers showed that as a screening tool in emergency department qSOFA needs significantly longer time than SIRS criteria for sepsis detection; thus, relying on qSOFA alone may delay initiation of interventions need for sepsis outcome improvement.^8^ This is in contrast to our results because we used an education course for our medical staff in emergency department regarding qSOFA warning alarms; moreover, we screened patients frequently. Therefore, we identified septic patients earlier compared to the similar trials. Results of JAAMSRA study showed that qSOFA had a suboptimal level for prediction in outside of ICU and could not identify 16% of patients with sepsis of whom 15.9% died.^23^ Our results showed that qSOFA could not detect sepsis in almost 35% but only 5% of them expired. The lower mortality rate in our trials can be due to early detection of sepsis and appropriate management of them accordingly.

### Limitations of the study

Our study was a single-center study with almost low sample size in a heterogenous critically ill patients group with infection in a tertiary university affiliated hospital. Thus, we need more trials with larger sample size for generalisability of the results. Strength of our study was implementation of an education course before study and emphasis on frequent screening of patients with suspected infection. The compliance of medical staff with the criteria was so high that lead to early detection and management of these patients which was the strength of our study.

## CONCLUSION

In patients with suspected sepsis, qSOFA has acceptable value for risk stratification of severity, multi organ failure and mortality. It seems that education of medical staff and frequent screening of patients for warning signs can help to increase the value of qSOFA in prediction of mortality in critically ill septic patients. Since the qSOFA criteria is an easy way of assessment with no need for laboratory tests, it can help the pre-hospital caregivers and ED clinicians to identify septic patients at a greater risk of poor outcome. We hope this small study will provoke more investigation into the appropriateness of fully adopting the qSOFA score as a sepsis screening tool by emergency medicine clinicians.

### Authors’ contribution

**KS:** Design of the study, reviewed the final draft.

**RJ:** Helped in data collection and analysis.

**PM:** Assisted in analysis and drafting.

**AM:** Performed the analyses and drafted the manuscript, takes responsibility for integrity of research.

All authors read and approved the final version of the manuscript.
